# Structure of GrlR and the Implication of Its EDED Motif in Mediating the Regulation of Type III Secretion System in EHEC

**DOI:** 10.1371/journal.ppat.0030069

**Published:** 2007-05-18

**Authors:** Chacko Jobichen, Mo Li, Gal Yerushalmi, Yih Wan Tan, Yu-Keung Mok, Ilan Rosenshine, Ka Yin Leung, J Sivaraman

**Affiliations:** 1 Department of Biological Sciences, National University of Singapore, Singapore; 2 Department of Molecular Genetics and Biotechnology, Faculty of Medicine, The Hebrew University, Jerusalem, Israel; The Rockefeller University, United States of America

## Abstract

Enterohemorrhagic Escherichia coli (EHEC) is a common cause of severe hemorrhagic colitis. EHEC's virulence is dependent upon a type III secretion system (TTSS) encoded by 41 genes. These genes are organized in several operons clustered in the locus of enterocyte effacement. Most of the locus of enterocyte effacement genes, including *grlA* and *grlR,* are positively regulated by Ler, and Ler expression is positively and negatively modulated by GrlA and GrlR, respectively. However, the molecular basis for the GrlA and GrlR activity is still elusive. We have determined the crystal structure of GrlR at 1.9 Å resolution. It consists of a typical β-barrel fold with eight β-strands containing an internal hydrophobic cavity and a plug-like loop on one side of the barrel. Strong hydrophobic interactions between the two β-barrels maintain the dimeric architecture of GrlR. Furthermore, a unique surface-exposed EDED (Glu-Asp-Glu-Asp) motif is identified to be critical for GrlA–GrlR interaction and for the repressive activity of GrlR. This study contributes a novel molecular insight into the mechanism of GrlR function.

## Author Summary

Attaching and effacing pathogens are a group of enteric pathogens that includes the closely related enterohemorrhagic Escherichia coli (EHEC) and enteropathogenic E. coli (EPEC). EPEC causes severe diarrhea in young children in developing countries, while EHEC is a causative agent of hemorrhagic colitis. A major infection mechanism employed by EHEC and EPEC is the type III secretion system (TTSS). TTSS is a syringe-like apparatus composed of approximately 20 proteins that serve to transfer virulence proteins from the bacteria directly into the host cytoplasm. The genes encoding for the TTSS components and related proteins are organized in several operons that are clustered in the locus of enterocyte effacement (LEE). GrlR and GrlA are LEE-encoded, newly identified, regulators that are common to all the attaching and effacing pathogens. This article reports the crystal structure of GrlR and explains how it can bind with GrlA to influence the activity of TTSS. Further, we have identified an EDED motif of GrlR crucial for the recognition of GrlA and activity. This study will help to understand the virulence determinants of *E. coli,* which is important for controlling the diseases caused by these organisms.

## Introduction

The enterohemorrhagic Escherichia coli (EHEC) and enteropathogenic E. coli (EPEC) are closely related human enteric pathogens [1]. EPEC causes severe diarrhea in young children in developing countries, while EHEC is a causative agent of hemorrhagic colitis, which is more common in the industrialized world [2]. EPEC, EHEC, and the mouse pathogen Citrobacter rodentium (CR) belong to a group of pathogenic bacteria that are defined by their ability to form “attaching and effacing” (AE) histopathology on intestinal epithelia. This histopathology is characterized by localized destruction of apical microvilli, followed by intimate adhesion of bacteria to the cell plasma membrane [3]. A major virulence mechanism underlying AE-causing bacteria is the type III secretion system (TTSS), which is employed by the bacteria as a molecular syringe to inject (translocate) effectors into the host cell. These effector proteins subvert normal host cell functions to benefit the bacteria [4–6]. TTSS components and related proteins are encoded by 41 genes organized in five major operons, *LEE1* through *LEE5*, and several additional transcriptional units, all clustered in the locus of enterocyte effacement (LEE) [7].

Under repressive conditions, the entire LEE is silenced by the histone-like nucleoid structuring protein (H-NS). Activation of most LEE promoters is dependent on Ler, an H-NS paralogue encoded by *LEE1,* which functions as anti–H-NS to alleviate the H-NS–mediated silencing of most of the LEE promoters [8–11]. Therefore, controlling the activity of the *LEE1* promoter (P_*LEE1*_) is critical for initiating a cascade that mediates the expression of all of the LEE genes. GrlA and GrlR are two LEE-encoded regulators that are required to optimize P_*LEE1*_ activity [12]. These two proteins from EPEC and EHEC, respectively, exhibit about 98% identities. GrlA acts as a positive regulator for P_*LEE1*_; moreover, GrlA and Ler form a positive transcriptional regulatory loop acting synergistically to strongly activate *ler* expression [12]. It is suggested that in order to prevent the detrimental accumulation of Ler, the Ler–GrlA feedback loop is negatively modulated by two checkpoints: (1) When Ler reaches the threshold concentration, it represses *ler* transcription [13]. (2) GrlR, a negative regulator of *ler* expression [14,15], might act as anti-GrlA to establish an additional checkpoint that down-regulates the feedback loop, setting it back to the steady-state level. In agreement with this hypothesis, GrlR interacts with itself and also with GrlA to form a macromolecular assembly in the cytoplasm of AE pathogens [16]. It has been proposed that GrlR conveys a negative regulation through its interaction with GrlA and that this hetero-complex is functionally relevant [12,16]. The literature search and sequence analysis indicated a presence of a helix-turn-helix, a DNA recognition motif at the N-terminus of GrlA [14], and the C-terminal region may interact with GrlR.

Here, we report the crystal structure of GrlR from EHEC refined up to 1.9 Å resolution as well as structure-based functional studies on GrlR. GrlR has a typical β-barrel fold consisting of an internal hydrophobic cavity with a plug-like loop on one side of the barrel. Structure-based site-directed mutagenesis targeting the surface residues of GrlR showed that these residues are crucial for the ability of GrlR to bind GrlA and to carry out its regulatory function. In vitro and in vivo experiments further confirmed the vital role of these residues for the regulatory function of GrlR. Our finding represents a novel regulatory mechanism in the TTSS of pathogenic bacteria.

## Results

### Structure of GrlR

**Figure 1:**
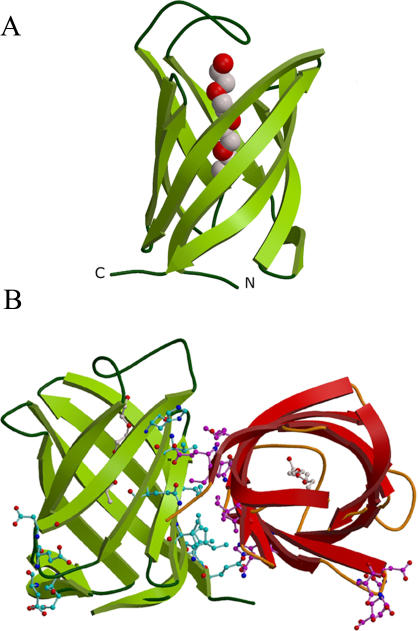
Overall Structure of GrlR with Bound Ligand (A) Ribbon diagram of monomer. The β-strands and the random coils/turns are depicted in light and dark green colors, respectively. The Triton-X100 molecule is bound in the hydrophobic pore of the eight-stranded β-barrel. The CPK model of the ligand is shown with oxygen and carbon atoms colored in red and grey, respectively. (B) Ribbon diagram of the dimer. Monomer A is shown in red, monomer B in green. The dimeric interface residues, surface-exposed residues (EDED motif), and the ligand from both monomers are shown in ball and stick representation. This figure was drawn using Molscript and Raster3D [42,43].

The structure of recombinant GrlR from EHEC was solved by the multi-wavelength anomalous dispersion method from synchrotron data. The model was refined to a final R-factor of 0.215 (R_free_ = 0.269) at 1.9 Å resolution (Figure 1A) with good stereochemical parameters (Table 1). The GrlR model consists of residues from Met1 to Val111, with seven additional residues at the N-terminus (Gly-6, Leu-5, Val-4, Pro-3, Arg-2, Gly-1, and Ser0) resulting from the linker sequence of the (His)_6_ affinity tag. The C-terminal residues from Asn112 to Lys121 had no interpretable electron density and were not modeled. There are two molecules in the asymmetric unit (Figure 1B) and they are related by a 2-fold noncrystallographic symmetry approximately parallel to the *a*-axis. Interestingly, these two molecules are packed in a perpendicular fashion to each other, resulting in a maximum interaction (Figure 1B).

**Table 1:**
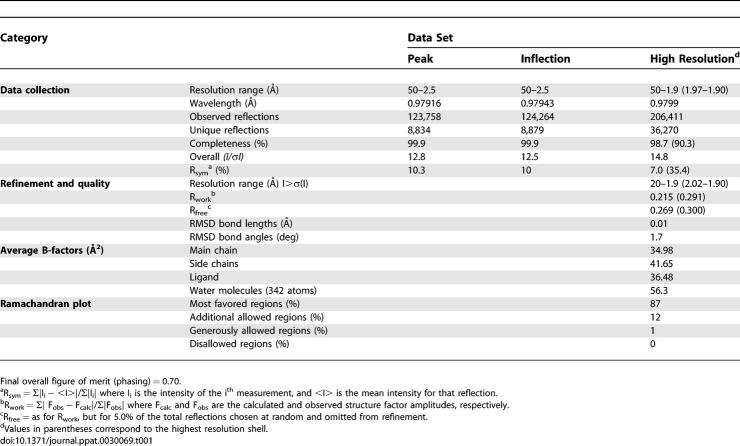
Crystallographic Data and Refinement Statistics

**Figure 2:**
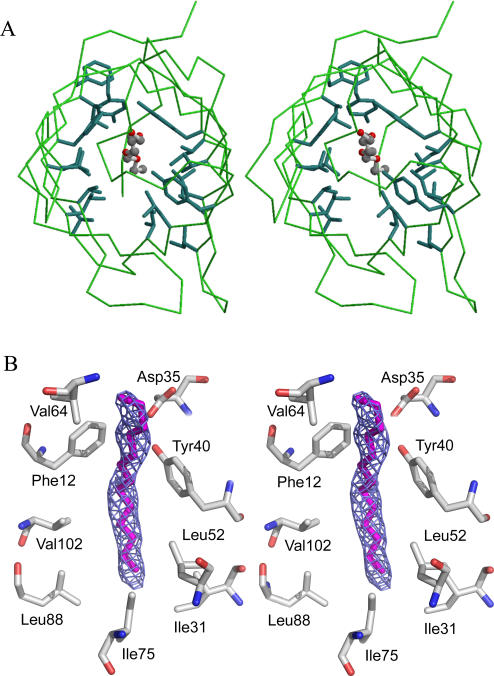
Stereo View of the β-Barrel and the Bound ligand (A) Stereo view of the Cα trace of GrlR β-barrel shown in green, viewed from the top. The hydrophobic side chains of the residues from the pore region are shown in thick lines. The ball-and-stick representation of the Triton-X100 molecule at the center of the pore is shown. This figure was prepared by using Molscript and Raster3D [42,43]. (B) Simulated annealing *Fo-Fc* omit map in the pore region of GrlR. The bound triton molecule and all atoms within 2 Å of the triton molecule were omitted prior to refinement. The map contoured at a level of 3σ. This figure was prepared using PyMOL [44].

The GrlR molecule mainly consists of a single domain from residues Asp5 to Ile107 that forms a β-barrel. Residues Tyr59 to Asp70 form an extended loop that plugs one side of the cylindrical β-barrel structure. The β-barrel consists of eight anti-parallel β-strands running from one side of the molecule to the other. On one side of the β-barrel there are four long loops, including a plug-like structure, whereas on the other side, four tight β-turns are connecting the adjacent β-strands. Both ends of the β-barrel were closed off by the N-terminus (Met1 to Lys4) and plug-like loop residues Tyr59 to Asp70. The tip of the ten residue–long plug-like loop, which is highly hydrophobic, may close or open the cavity primarily by hydrophobic interactions. The β-barrel cavity is highly hydrophobic in nature with side chains from seven Tyr, six Ile, seven Leu, four Val, and two Phe residues lining the inner cavity surface (Figure 2A). The approximate dimensions of the β-barrel are 35.2 Å in height and 18.5 Å in diameter.

GrlR shares 93% to 100% identity between different AE-causing E. coli strains and EHEC (strain EDL933) and over 85% sequence identity (CLUSTAL W [17]) with that of CR. There is no significant overall sequence identity with any other protein in the National Center for Biotechnology Information (NCBI) database. A search for topologically similar proteins within the Protein Data Bank (PDB) database was performed with the program DALI [18]. The highest structural homology is observed between GrlR and the electron transport domain of quinohemoprotein amine dehydrogenase (PDB code 1jju; with 18% sequence identity; *z*-score 9.2 and 2.5 Å RMSD [root mean square deviation] for 90 Cα atoms). This is followed by a lipid-binding TTSS secretin pilotin protein, MxiM (PDB code 1y9t; with 16% sequence identity; *z*-score 4.90 and 2.7 Å RMSD for 77 Cα atoms), which has a cracked barrel structure [19].

During structure refinement, we noticed a small molecule composed of 12 atoms in the hydrophobic cavity of GrlR, which, subsequently, was identified to be the fragment of Triton-X100. It is worth mentioning here that the bacterial lysis buffer used for GrlR purification contained 1% (v/v) of Triton-X100. The detergent may have bound tightly to the hydrophobic cavity of GrlR during this stage, and had co-crystallized with GrlR. The bound detergent is situated at the center of the cavity and is parallel to its axis (Figure 1A); the interaction of the detergent with hydrophobic residues of the cavity may play a crucial role in increasing the solubility of GrlR (Figure 2A). The ligand molecule is well defined in the electron density map. Figure 2B shows the simulated annealing Fo-Fc omit map. The superimposed GrlR on lipid-bound MxiM [19] indicated that the probability of having a lipid molecule in the hydrophobic pore of GrlR is not ruled out. Based on the structural homology, the bound ligand, and the hydrophobic nature of the cavity of GrlR, we suggest that the cavity may recognize a specific small hydrophobic ligand and interact with side chains of the cavity residues. Exact roles of this cavity and the plug-like loop for the function of GrlR are not yet established.

### Dimers of GrlR

**Figure 3:**
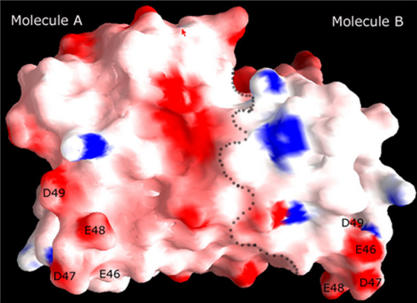
The Electrostatic Surface Potential of GrlR Dimer The surface-exposed (EDED) residues are labeled. It shows a strong negatively charged (red) patch from both EDED motifs of the monomers. The dotted line indicates the dimer interface. Blue represents a positive charge. This figure was prepared using GRASP [20].

GrlR was found to exist as a homodimer in solution, with an apparent molecular weight of 29 kDa, as determined by gel filtration and dynamic light scattering. The analytical ultra centrifugation experiments also revealed the dimeric nature of GrlR. These results were consistent with a dimeric arrangement observed in the crystal structure, with the dimer having approximate dimensions of 46.5 × 32.6 × 35.2 Å. The strong hydrophobic cluster at the dimeric interface is maintained by the side chains from residues Ile7, Ile23, Ile25, Val39, and Ile107 from both monomers of the dimer. In addition, six hydrogen bonding contacts (<3.2 Å) mainly from Gln41, Glu108, His55, Pro109, and Val 111 of both monomers, as well as numerous hydrophobic interactions, are maintaining the dimer architecture. Figure 3 shows the electrostatic surface representation of the dimeric GrlR (GRASP [20]). The observation of a dimeric GrlR for the wild-type as well as for mutants suggests a functionally important role for dimerization.

### Identification of a Key Motif

GrlR was shown to associate with itself and with GrlA to mediate the regulatory network [12,17]. GrlA is homologous to CaiF, a known DNA binding protein [21], and its sequence analysis identified a helix-turn-helix DNA recognition motif at the N-terminus [12]. The C-terminal region of GrlA is rich in basic residues (nine arginines and seven lysines), suggesting that it may have a role in the interaction with the acidic GrlR. A loop region in the crystal structure of GrlR, residues Glu46 to Asp49 (^46^EDED^49^), is highly exposed on the surface and is less well defined in electron density. It is worth noting here that GrlA and GrlR have extremely opposite charges: the calculated isoelectric points (pI) of GrlR and GrlA are 4.83 and 9.71, respectively. Taking together all of these facts, we now hypothesize that the negatively charged cluster (the EDED [Glu-Asp-Glu-Asp] motif) is involved in the GrlR–GrlA interaction and thus may play an important role for repressing the ability of EHEC to perform TTSS-mediated protein secretion.

### EDED Motif Is Essential for the Recognition of GrlA

**Figure 4:**
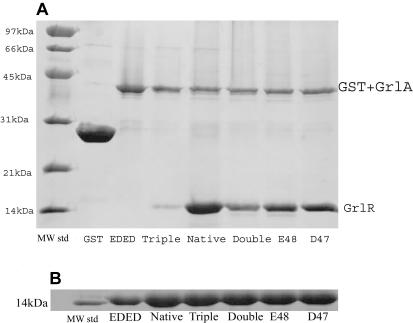
In Vitro Pull-Down Assay (A) SDS-PAGE gel showing the in vitro pull-down assay to demonstrate the binding of wild-type GrlR and EDED mutants to GrlA. Lane 1, molecular weight marker (MW); lane 2, GST; lane 3, E46A-D47A-E48A-D49A; lane 4, E46A-D47A-E48A; lane 5, wild-type GrlR; lane 6, E46A-D47A; lane 7, E48A; lane 8, D47A. The bands corresponding to GST-GrlA and GrlR were further identified by peptide mass finger printing. The staining was done using coomassie brilliant blue. (B) SDS-PAGE gel showing the amount of native and mutant GrlR protein used for the in vitro pull-down assay. Lane 1, molecular weight marker; lane 2, E46A-D47A-E48A-D49A; lane 3, wild-type GrlR; lane 4, E46A-D47A-E48A; lane 5, E46A-D47A; lane 6, E48A; lane 7, D47A.

To test the above hypothesis, EDED residues were mutated and interactions of different GrlR mutants with glutathione-S-transferase (GST)–GrlA were tested by pull-down assays followed by SDS-PAGE analyses. We found that GrlA was not stable on its own, but the GrlA fused with GST was sufficiently stable for pull-down experiments with both wild-type and mutants of GrlR to verify the binding between these two proteins. We have also confirmed that wild-type GrlR binds to GST-GrlA (Figure 4A), whereas no binding has been observed with GST alone. All of the four single mutants (E46A, D47A, E48A, and D49A) and the double mutant (E46A-D47A) of GrlR showed similar binding to GrlA in pull-down assays. However, the triple mutant (E46A-D47A-E48A) showed a significant drop in binding to GrlA. Whereas the EDED tetra mutant, with all of the four residues mutated to alanine, did not bind to GrlA, no protein band corresponding to GrlR was detected in the pull-down assay (Figure 4A). Bands corresponding to GST-fused GrlA and GrlR were analyzed with peptide mass finger printing and their identities were confirmed (unpublished data). To verify the integrity of secondary structures in the mutants, circular dichroism spectra were measured for wild-type GrlR as well as for all mutants. In all cases, the circular dichroism spectra showed the existence of similar β-sheet secondary structures, which is consistent with the crystal structure. These results indicated that the surface-exposed EDED motif is a key structural feature for the binding of GrlR to GrlA.

### Regulatory Function of GrlR Is Mediated by EDED Motif

**Figure 5:**
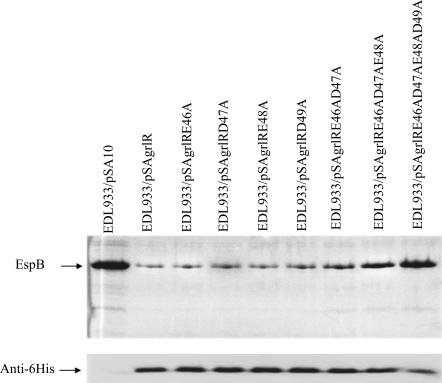
General Secretion Profile of EHEC EDL933 Harboring pSA10-*grlR* and Expressing Wild-Type and Mutants of GrlR Secreted proteins were concentrated from supernatants of bacterial culture grown in DMEM and resolved in 12% SDS-PAGE and stained with coomassie brilliant blue (upper panel). Western blot of EHEC total cell protein against 6His antibody (lower panel).

In order to elucidate the role of the wild-type and mutants of GrlR in the repression of TTSS, a protein secretion assay was carried out in EHEC. Bacteria containing different plasmids were grown in Dulbecco's Modified Eagle's Medium (DMEM), and total secreted extracellular proteins were recovered from the medium and compared by SDS-PAGE. We used EspB, which is a major secreted protein of EHEC, as a representative marker and compared the secretion of EspB in wild-type and mutant GrlR (Figure 5, upper panel). The wild-type GrlR repressed the secretion of EspB. Single (E46A, D47A, E48A, D49A) and double mutants (E46A-D47A) showed similar reduced secretion of this protein, whereas the triple mutant (E46A-D47A-E48A) significantly reduced GrlR repression and the secretion of EspB was increased. However, the tetra mutant (E46A-D47A-E48A-D49A) totally abolished the repression effect of GrlR and the secretion was restored to a level similar to that of the control. We have also verified the expression level of all of the GrlR mutants (Figure 5, lower panel). These results are consistent with our previous pull-down assays (Figure 4A), which showed that GrlR and GrlA binding was affected in the triple and tetra mutants. Our experiment demonstrated the importance of the EDED motif for carrying out the regulatory function of TTSS in EHEC.

### EDED Motif Is Required for Repression of P_*LEE1*_ In Vivo

A global regulator of TTSS, Ler, which is encoded by the first gene of *LEE1*, positively regulates several secreted proteins, including EspB [9,22]. It has been shown that GrlR overexpression suppresses production of Ler [12,14]. From our experiments, we have shown that overexpression of wild-type GrlR, but not mutated GrlR, affects the secretion of EspB.

The role of the EDED motif in repressing the activity of the P_*LEE1*_ was analyzed by a transcription kinetics assay. Since the signal generated by a single copy chromosomal *gfp* gene under the P_*LEE1*_ is too low to detect in EHEC due to intrinsic low expression levels of the *LEE1* in EHEC, we performed our studies in the closely related EPEC, where the intrinsic level of *LEE1* expression is higher. To this end, we have constructed an EPEC strain (GY2455) expressing GFP+ (green fluorescent protein) from the native *LEE1* promoter (P*_LEE1_*). The fluorescence (amount of GFP) as well as OD_600_ (amount of bacteria) were determined, in real time, during growth. The presence of plasmid encoding GrlR did not affect *gfp* expression unless IPTG was added. Under our experimental conditions, GrlR expression resulted in attenuation of P_*LEE1*_ activity, but not complete repression. Similar results were seen with the plasmids encoding GrlR, which mutated at different residues of the EDED motif. Upon replacing the entire EDED motif with AAAA, GrlR was no longer capable of P_*LEE1*_ repression (Figure S2). These results support the hypothesis that the EDED motif is crucial for repression of P_*LEE1*_ by GrlR. Overall, results of this *gfp* assay, with the exception of small variations, are comparable to EHEC experiments. These observed variations may be due to the difference in strains as well as the experimental conditions.

## Discussion

The regulatory network that controls the expression of the virulence genes of AE pathogens is complex. Much of this complexity is merged at controlling the activity of the P_*LEE1*_ and *ler* expression. We, as well as other groups, demonstrated that Ler is a master regulator, turning on and off a large number of virulence genes, including *espB* [9,22,23]. The punctually temporal regulation of Ler and maintaining its accurate levels of activity are essential for the successful colonization of the host [12,16]. The GrlR−GrlA complex plays a key role in controlling Ler expression [12,14]. Iyoda et al. [24] reported recently that the GrlR–GrlA complex also controls the expression of FlhDC, the flagella master regulator. Thus, the GrlR–GrlA complex plays an important role in controlling the expression of two key master regulators, Ler and FlhDC. Structural study of GrlR identified the surface-exposed EDED motif and the importance of these residues was further investigated. Our in vivo and in vitro functional studies of wild-type and mutant GrlR showed that the EDED motif is crucial for the recognition of GrlA by GrlR and for the GrlR regulatory activity. To our knowledge, this is the first report of a key role of the EDED motif in bacterial regulation.

Based on the properties of these two proteins, the location of the EDED motif in the dimeric GrlR and the dimeric nature of most of the helix-turn-helix–containing DNA-binding proteins, we propose that the GrlA may also exist as a homodimer, and that dimers of GrlR and GrlA in combination are involved in the regulatory mechanism*.*

Our study provides a novel structural basis for an understanding of the regulatory mechanism of the GrlA–GrlR complex and thus provides new insight into the complex regulatory network that governs the virulence of AE pathogens.

## Materials and Methods

### Plasmid and strain construction.

The bacterial stains and the plasmids used in this study are listed in Table S1. The intact *grlR* gene was PCR amplified from EHEC EDL933 chromosomal DNA and cloned into a derivative of pET vector (pETM32) (Novagen, http://www.emdbiosciences.com/html/NVG/home.html) or pSA10 vector. The respective targeted residues were substituted with alanine. Plasmid pGEX-*grlA* was constructed by amplifying the *grlA* DNA fragments from EHEC EDL933 chromosomal DNA and cloning into pGEX-4T1 (Amersham Biosciences, http://www.gelifesciences.com). Construction of pGY2Ler was as follows. The *bla* gene in the suicide plasmid pGP704 [25] was replaced by *tetAR,* and the *gfp+* gene [26] was cloned into the XbaI and SmaI sites generating pGY2. A fragment containing P*_LEE1-ler_* (the regulatory region of *LEE1* and the first gene in *LEE1-ler*) was amplified by PCR, digested with BamHI and XbaI, and cloned into pGY2 digested with BglII and XbaI, generating pGY2Ler, in which *gfp+* is transcriptionally fused to *ler*. pGY2Ler was introduced into E. coli SM10 λpir, which was further introduced into EPEC by mating. A trans-conjugant Kan^S^ Tet^R^ Strep^R^ colony containing an integration of pGY2Ler into the EPEC chromosome was selected to form transcriptional fusion of the *LEE1* regulatory region with *ler* and the *gfp+* gene (Figure S1) and was termed strain GY2455.

### Purification and crystallization.

Plasmid DNA was transformed into E. coli BL21 and the cells were grown in defined M9 medium [27] supplemented with 25 mg/l L-SeMet at 37 °C to 0.6 AU at OD_600._ One liter of culture was induced with 100 μM IPTG and continued to grow at 20 °C overnight. Cells were then harvested by centrifugation and resuspended in 40 ml of lysis buffer (50 mM Tris-HCL [pH 7.5], 0.4 M NaCl, 1 mM EDTA, 1% (w/v) Triton X-100, 5% (w/v) glycerol, 10 mM ßME, and one tablet of Complete protease inhibitors [Roche Diagnostics, http://www.roche.com]). The protein was purified in three steps using DEAE-Sepharose (Amersham Pharmacia, http://www.gelifesciences.com), NI-NTA (Qiagen, http://www.qiagen.com), and gel filtration (Superdex 75, Amersham Biosciences) columns, respectively. The His fusion tag was not cleaved. Drops containing 1 μl of protein solution (4 mg/ml) and 1 μl of reservoir solution were equilibrated by hanging drop vapor diffusion at 21 °C. The best crystals were grown from 25% ethylene glycol, 4% tert-butanol, and 4% trifluoroethanol (Hampton screens followed by additive screening), with the protein in 20 mM Tris-HCL (pH 7.5), 200 mM NaCl, and 5% (w/v) glycerol. Crystals measuring ~0.2 mm in length grown over the course of 3 d belonged to space group P2_1_2_1_2_1_ with a = 43.83 Å, b = 66.09 Å, c = 83.58 Å and contained two molecules in the asymmetric unit. The Matthews coefficient is 2.2 Å^3^/Da [28], giving a solvent content of 45%. The X-ray data collection and refinement statistics are given in Table 1.

### Data collection, structure solution, and refinement.

Crystals were cryoprotected in the reservoir solution supplemented with 40% ethylene glycol and flash cooled at 100 K. The structure was determined using crystals of SeMet-labeled protein by multi-wavelength anomalous dispersion method [29]. X-ray diffraction data were collected at beamline X12C, Brookhaven National Laboratory (Upton, New York, United States), using a Quantum-4 CCD detector (ADSC, http://www.adsc-xray.com). Two data sets were collected at wavelengths corresponding to the peak and inflection point. All of the data sets were processed with HKL2000 [30]. All of the eight Se sites of an asymmetric unit were located by using the program BnP [31]. The phases were further improved by density modification using RESOLVE [32], which gave a final overall figure of merit of 0.70. Over 50% of the backbone atoms of the model were built by the RESOLVE iteration method. The remaining residues of the molecules were added after several cycles of manual model building using O [33] and followed by refinement using CNS [34]. Finally, 342 well-defined water molecules were added, and refinement was continued until the R-value converged to 0.215 (R_free_ = 0.269) for reflections I>σ(I) to 1.9 Å resolution. The model had good stereochemistry, with all residues falling within the allowed regions of the Ramachandran plot (Table 1) analyzed by PROCHECK [35].

### In vitro pull-down assay.

The plasmid pGEX-*grlA* was transformed into E. coli strain BL21 DE3 and overexpressed under IPTG induction. GrlA protein with GST tag was immobilized on GST sepharose beads (Amersham Biosciences) in Lysis buffer (20 mM TRIS [pH 7.5], 200 mM NaCl, 5% glycerol, 10 mM ßME ) and washed with wash buffer at various salt concentrations (200 mM, 500 mM, and 1 M NaCl) to remove non-specific bound protein from the beads. The beads with immobilized GrlA protein were checked for purity and quantified using SDS-PAGE and were subsequently used for performing the pull-down assay studies. Expression and purification of his-tagged GrlR fusion proteins were performed as described previously. Equal amounts of GrlR wild-type and mutant proteins were added to the GST sepharose beads with bound GrlA and incubated at 4 °C for 15 min (Figure 4B). The expression level of GrlR tetra mutant was low compared to that of the wild-type and other mutants; however, an approximately equal amount of tetra mutant was used in all of the experiments. The beads were washed twice with wash buffer and resolved in 12% SDS-PAGE along with the controls.

### Analytical ultra centrifugation.

The oligomeric state of GrlR was investigated by monitoring its sedimentation properties in sedimentation velocity experiments using a Beckman Coulter Optima XL-A (http://www.beckmancoulter.com) equipped with absorbance optics. Sedimentation coefficients and molecular masses were determined by fitting using both the C(s) method [36] and Enhanced van Holde–Weischet Analysis [37] as implemented in UltraScan 7.3 [38,39].

### MALDI-TOF MS and MS/MS analysis.

Molecular weight determination was carried out with the aid of a Voyager STR MALDI-TOF mass spectrometer (Applied Biosystems, http://www.appliedbiosystems.com). For MS/MS analysis, sample digestion, desalting, and concentration steps were carried out by using the Montage In-Gel digestion Kits (Millipore, http://www.millipore.com). Protein spots were analyzed using an Applied Biosystems 4700 Proteomics Analyzer MALDI-TOF/TOF (Applied Biosystems). Data processing and interpretation was carried out using the GPS Explorer software (Applied Biosystems) and database searching was performed using the MASCOT program (Matrix Science, http://www.matrixscience.com). The National Center for Biotechnology Information database (http://www.ncbi.nlm.nih.gov) was used for the combined MS and MS/MS search.

### Circular dichroism spectrometry.

Far UV spectra (260–190 nm) of GrlR wild-type and mutants were measured using Jasco J810 spectropolarimeter (Jasco, http://www.jascoinc.com) in phosphate buffer (pH 7.5) at room temperature using 0.1 cm path length, stoppered cuvettes. A total of three scans were recorded and averaged for each spectrum, and the baseline was subtracted.

### Extracellular proteins isolation and assay.

To prepare the secreted protein of EHEC, overnight cultures of EHEC strains in LB were diluted at 1:50 into DMEM supplemented with 100 mM ampicillin and 0.1 mM IPTG, and incubated for 9 h at 37 °C in a 5% (v/v) CO_2_ atmosphere. Bacterial cells were removed from the culture by centrifugation (5,500*g,* 10 min, 4 °C) and the supernatants were collected and passed through a 0.22-μm filter and precipitated by 10% TCA as described previously [22]. The extracellular proteins were resolved in 12% SDS-PAGE and visualized by staining with commassie blue. Western blot analysis was carried out as described previously [40], and EHEC cells harboring various pSA*grlR* plasmids were harvested and resolved by SDS-PAGE. Proteins were transferred to PVDF membrane and detected by anti-6His (Qiagen) antibody.

### Measurement of the P_*LEE1*_ activity in vivo.

Plasmids (pSA10) expressing GrlR or various GrlR mutants from the *Ptac* promoter were introduced into EPEC GY2455. The generated strains were grown overnight under conditions that repress the activity of the P_*LEE1*_ (30 °C in LB) [26]. For activation of the P_*LEE1*_, the cultures were washed and 50 times diluted with Casamino-DMEM [26] supplemented or not supplemented with 0.25 mM IPTG. Immediately upon dilution, cultures, in 96-well plates, were placed in a microplate reader (SPECTRAFluor Plus; TECAN, http://www.tecan.com), pre-set at 37 °C and grown within the plate reader. The fluorescence intensity (filter set at 485-nm excitation wavelength and 535-nm emission wavelength) and optical densities at 600 nm (OD_600_) were automatically read during growth every 5 min, and data were collected by Magellan software (TECAN).

## Supporting Information

Figure S1 The Construction of an EPEC Strain Expressing GFP+ from the P_*LEE1*_
Plasmid pGY2Ler was introduced into EPEC via mating with E. coli SM10 λ*pir*. Bacteria containing integration of the plasmid to the chromosome were selected using LB plates supplemented with Tet and Strep. In these bacteria, one *ler* allele and *gfp+* are regulated by the native P_*LEE1*_ and the second *ler* allele, and other *LEE1* genes are expressed via a second native P*_LEE1_*. The latter is transcriptionaly isolated from the integrated plasmid by a terminator (T1) and by TetR, which represses the *tetRA* promoters under our experimental conditions (lack of Tet).
(5 MB TIF)

Figure S2 P_*LEE1*_ Activity Assay The presence of plasmid encoding GrlR did not affect the *gfp* expression unless IPTG was added (right panel). Replacing the entire EDED motif with AAAA, GrlR was no longer capable of P_*LEE1*_ repression (left panel). The EPEC strain containing fusion of the P_*LEE1*_ with *gfp*+ transcriptional reporter gene (GY2455) was transformed with different GrlR-expressing plasmids. These include plasmids expressing wild-type GrlR as positive control, the vector plasmid (pSA10) as negative control, and plasmids expressing different GrlR mutants. Expression of the *LEE1* was induced by shifting the culture from growth in LB at 30 °C to growth at 37 °C in CDMEM (supplemented with IPTG to induce GrlR expression). The rate of GFP accumulation in the bacteria reflecting the rate of promoter activity was determined. The fluorescence intensity (filter set at 485-nm excitation wavelength and 535-nm emission wavelength) and optical densities at 600 nm (OD_600_) were automatically read during growth every 5 min, and data were collected by Magellan software (TECAN). Shown are the results of a typical experiment out of three independent experiments. The wild-type GrlR, as well as all of the mutants, excluding the AAAA mutant, attenuated the activity rate of the P_*LEE1*_. In contrast, the AAAA mutant exhibits *LEE1* activity similar to that of a strain which is not expressing GrlR (control vector).
(4.9 MB TIF)

Table S1 The Bacterial Strains and Plasmids Used in This Study (45 KB DOC)

### Accession Number

Coordinates of GrlR have been deposited in the Protein Data Bank (http://www.pdb.org [41]) under accession code 2OVS.

## Abbreviations

AE: attaching and effacing
CR: Citrobacter rodentium
DMEM: Dulbecco's Modified Eagle's Medium
EHEC: enterohemorrhagic Escherichia coli
EPEC: enteropathogenic Escherichia coli
GFP: green fluorescent protein
GST: glutathione-S-transferase
H-NS: histone-like nucleoid structuring protein
LEE: locus of enterocyte effacement
P_*LEE1*_: *LEE1* promoter
RMSD: root mean square deviation
TTSS: type III secretion system

## References

[ppat-0030069-b001] Polotsky YE, Dragunskaya EM, Seliverstova VG, Avdeeva TA, Chakhutinskaya MG (1977). Pathogenic effect of enterotoxigenic Escherichia coli and Escherichia coli causing infantile diarrhoea. Acta Microbiol Acad Sci Hung.

[ppat-0030069-b002] Nataro JP, Kaper JB (1998). Diarrheagenic Escherichia coli. Clin Microbiol Rev.

[ppat-0030069-b003] Paton AW, Manning PA, Woodrow MC, Paton JC (1998). Translocated intimin receptors (Tir) of Shiga-toxigenic Escherichia coli isolates belonging to serogroups O26, O111, and O157 react with sera from patients with hemolytic-uremic syndrome and exhibit marked sequence heterogeneity. Infect Immun.

[ppat-0030069-b004] Caron J, Lariviere L, Nacache M, Tam M, Stevenson MM (2006). Influence of Slc11a1 on the outcome of Salmonella enterica serovar Enteritidis infection in mice is associated with Th polarization. Infect Immun.

[ppat-0030069-b005] Hayward RD, Leong JM, Koronakis V, Campellone KG (2006). Exploiting pathogenic Escherichia coli to model transmembrane receptor signalling. Nat Rev Microbiol.

[ppat-0030069-b006] Spears KJ, Roe AJ, Gally DL (2006). A comparison of enteropathogenic and enterohaemorrhagic Escherichia coli pathogenesis. FEMS Microbiol Lett.

[ppat-0030069-b007] Clarke SC, Haigh RD, Freestone PP, Williams PH (2003). Virulence of enteropathogenic *Escherichia coli,* a global pathogen. Clin Microbiol Rev.

[ppat-0030069-b008] Friedberg D, Umanski T, Fang Y, Rosenshine I (1999). Hierarchy in the expression of the locus of enterocyte effacement genes of enteropathogenic Escherichia coli. Mol Microbiol.

[ppat-0030069-b009] Mellies JL, Elliott SJ, Sperandio V, Donnenberg MS, Kaper JB (1999). The Per regulon of enteropathogenic *Escherichia coli:* Identification of a regulatory cascade and a novel transcriptional activator, the locus of enterocyte effacement (LEE)-encoded regulator (Ler). Mol Microbiol.

[ppat-0030069-b010] Sperandio V, Mellies JL, Delahay RM, Frankel G, Crawford JA (2000). Activation of enteropathogenic Escherichia coli (EPEC) LEE2 and LEE3 operons by Ler. Mol Microbiol.

[ppat-0030069-b011] Sanchez-SanMartin C, Bustamante VH, Calva E, Puente JL (2001). Transcriptional regulation of the orf19 gene and the tir-cesT-eae operon of enteropathogenic Escherichia coli. J Bacteriol.

[ppat-0030069-b012] Barba J, Bustamante VH, Flores-Valdez MA, Deng W, Finlay BB (2005). A positive regulatory loop controls expression of the locus of enterocyte effacement-encoded regulators Ler and GrlA. J Bacteriol.

[ppat-0030069-b013] Berdichevsky T, Friedberg D, Nadler C, Rokney A, Oppenheim A (2005). Ler is a negative autoregulator of the LEE1 operon in enteropathogenic Escherichia coli. J Bacteriol.

[ppat-0030069-b014] Deng W, Puente JL, Gruenheid S, Li Y, Vallance BA (2004). Dissecting virulence: Systematic and functional analyses of a pathogenicity island. Proc Natl Acad Sci USA.

[ppat-0030069-b015] Lio JC, Syu WJ (2004). Identification of a negative regulator for the pathogenicity island of enterohemorrhagic Escherichia coli O157:H7. J Biomed Sci.

[ppat-0030069-b016] Creasey EA, Delahay RM, Daniell SJ, Frankel G (2003). Yeast two-hybrid system survey of interactions between LEE-encoded proteins of enteropathogenic Escherichia coli. Microbiology.

[ppat-0030069-b017] Thompson JD, Higgins DG, Gibson TJ (1994). CLUSTAL W: Improving the sensitivity of progressive multiple sequence alignment through sequence weighting, position-specific gap penalties and weight matrix choice. Nucleic Acids Res.

[ppat-0030069-b018] Holm L, Sander C (1998). Touring protein fold space with Dali/FSSP. Nucleic Acids Res.

[ppat-0030069-b019] Lario PI, Pfuetzner RA, Frey EA, Creagh L, Haynes C, Maurelli AT, Strynadka NC (2005). Structure and biochemical analysis of a secretin pilot protein. EMBO J.

[ppat-0030069-b020] Nicholls A, Sharp KA, Honig B (1991). Protein folding and association: Insights from the interfacial and thermodynamic properties of hydrocarbons. Proteins.

[ppat-0030069-b021] Buchet A, Nasser W, Eichler K, Mandrand-Berthelot MA (1999). Positive co-regulation of the Escherichia coli carnitine pathway *cai* and *fix* operons by CRP and the CaiF activator. Mol Microbiol.

[ppat-0030069-b022] Li M, Rosenshine I, Tung SL, Wang XH, Friedberg D (2004). Comparative proteomic analysis of extracellular proteins of enterohemorrhagic and enteropathogenic Escherichia coli strains and their ihf and ler mutants. Appl Environ Microbiol.

[ppat-0030069-b023] Iyoda S, Watanabe H (2004). Positive effects of multiple pch genes on expression of the locus of enterocyte effacement genes and adherence of enterohaemorrhagic Escherichia coli O157 : H7 to HEp-2 cells. Microbiology.

[ppat-0030069-b024] Iyoda S, Koizumi N, Satou H, Lu Y, Saitoh T (2006). The GrlR-GrlA regulatory system coordinately controls the expression of flagellar and LEE-encoded type III protein secretion systems in enterohemorrhagic Escherichia coli. J Bacteriol.

[ppat-0030069-b025] Donnenberg MS, Kaper JB (1991). Construction of an eae deletion mutant of enteropathogenic Escherichia coli by using a positive-selection suicide vector. Infect Immun.

[ppat-0030069-b026] Hautefort I, Proenca MJ, Hinton JC (2003). Single-copy green fluorescent protein gene fusions allow accurate measurement of *Salmonella* gene expression in vitro and during infection of mammalian cells. Appl Environ Microbiol.

[ppat-0030069-b027] Doublie S (1997). Preparation of selenomethionyl proteins for phase determination. Methods Enzymol.

[ppat-0030069-b028] Matthews B W (1968). Solvent content of protein crystals. J Mol Biol.

[ppat-0030069-b029] Terwilliger TC, Berendzen J (1997). Bayesian correlated MAD phasing. Acta Cryst D.

[ppat-0030069-b030] Otwinowski Z, Minor W (1997). Processing of X-ray diffraction data collected in oscillation mode. Methods Enzymol.

[ppat-0030069-b031] Weeks CM, Blessing RH, Miller R, Mungee R, Potter SA (2002). Towards automated protein structure determination: BnP, the snB- PHASES interface. ZKristallogr.

[ppat-0030069-b032] Terwilliger TC (2003). SOLVE and RESOLVE: Automated structure solution and density modification. Methods Enzymol.

[ppat-0030069-b033] Jones TA, Zou JY, Cowan SW, Kjeldgaard M (1991). Improved methods for building protein models in electron-density maps and the location of errors in these models. Acta Crystallogr A.

[ppat-0030069-b034] Brunger AT, Adams PD, Clore GM, DeLano WL, Gros P (1998). Crystallography & NMR system: A new software suite for macromolecular structure determination. Acta Crystallogr D Biol Crystallogr.

[ppat-0030069-b035] Laskowski RA, MacArthur MW, Moss DS, Thornton JM (1993). PROCHECK: A program to check the stereochemical quality of protein structures. J App Cryst.

[ppat-0030069-b036] Schuck P (2000). Size-distribution analysis of macromolecules by sedimentation velocity ultracentrifugation and lamm equation modeling. Biophys J.

[ppat-0030069-b037] Van Holde KE, Weischet WO (1978). Boundary analysis of sedimentation velocity experiments with monodisperse and paucidisperse solutes. Biopolymers.

[ppat-0030069-b038] Demeler B, Saber H, Hansen JC (1997). Identification and interpretation of complexity in sedimentation velocity boundaries. Biophys J.

[ppat-0030069-b039] Demeler B (2005). UltraScan software.

[ppat-0030069-b040] Li M, Rosenshine I, Yu HB, Nadler C, Mills E, Hew CL (2006). Identification and characterization of NleI, a new non-LEE-encoded effector of enteropathogenic Escherichia coli (EPEC). Microbes Infect.

[ppat-0030069-b041] Berman HM, Westbrook J, Feng Z, Gilliland G, Bhat TN (2000). The Protein Data Bank. Nucleic Acids Res.

[ppat-0030069-b042] Kraulis PJ (1991). MOLSCRIPT: A program to produce both detailed and schematic plots of protein structures. J Appl Cryst.

[ppat-0030069-b043] Merrit EA, Bacon DJ (1997). Raster3d. Photorealistic molecular graphics. Methods Enzymol.

[ppat-0030069-b044] DeLano WL (2002). The PyMOL molecular graphics system.

