# Supplementary material for: Structure of GrlR and the Implication of Its EDED Motif in Mediating the Regulation of Type III Secretion System in EHEC
Source: PLoS Pathog. 2007 May 18;3(5):e69. doi: 10.1371/journal.ppat.0030069 (PMC1868957; doi:10.1371/journal.ppat.0030069)
Supplement: Supplementary file 1 [file ppat.0030069.st001.doc]

Table S1: The bacteria strains and plasmids used in this study

| Strain or plasmid | Description | Reference or source |
| --- | --- | --- |
| Strains |  |  |
| EDL933 | Wild-type EHEC O157:H7, Slt1, Slt2-negative derivative | J. Leong |
| E2348/69 | Prototypic EPEC strain, O127:H6, Sm^r^ | J. Kaper |
| SM10 λpir | *thi1 thr leu tonA lacY recA supE* (RP4-2 Km^r^ *tet*::Mu) | [1] |
| GY2455 | E2348/69 *LEE1reg-ler::gfp+* | This study |
| Plasmids |  |  |
| pGEX4T-1 | pGEX-2T derivative, Amp^r^ | Pharmacia |
| pGEX*grlA* | pGEX4T-1 with *grlA* fragment | This study |
| pSA10 | pKK177-3 derivative, *lacI*, Amp^r^ | S. Altuvia |
| pSA*grlR-6His* | pSA10 with *grlR*  fragment and 6His tag at C terminal, | This study |
| pSA*grlR* E46A | pSA10 with *grlR*  fragment and 6His tag at C terminal, substitute residue E46 with A | This study |
| pSA*grlR* D47A | pSA10 with *grlR*  fragment and 6His tag at C terminal, substitute residue D47 with A | This study |
| pSA*grlR* E48A | pSA10 with *grlR*  fragment and 6His tag at C terminal, substitute residue E48 with A | This study |
| pSA*grlR* D49A | pSA10 with *grlR*  fragment and 6His tag at C terminal, substitute residue D49 with A | This study |
| pSA*grlR* E46AD47A | pSA10 with *grlR*  fragment and 6His tag at C terminal, substitute residues E46D47 with AA | This study |
| pSA*grlR* E46AD47AE48A | pSA10 with *grlR*  fragment and 6His tag at C terminal, substitute residues E46D47E48 with AAA | This study |
| pSA*grlR* E46AD47AE48AD49A | pSA10 with *grlR*  fragment and 6His tag at C terminal, substitute residues E46D47E48D49 with AAAA | This study |
| pGP704 | *pir-*dependent, suicide plasmid | [2] |
| pGY2 | A pGP704 derivative in which *bla* was replaced by *tetAR* and containing *gfp+* as transcriptional reporter gene | This study |
| pGY2Ler | pGY2 encoding P*_LEE1_*_-_*_ler_* transcriptionally fused to the *gfp+* gene | This study |

**References**

1. Donnenberg MS, Kaper JB (1991) Construction of an eae deletion mutant of enteropathogenic *Escherichia coli* by using a positive-selection suicide vector. Infect Immun 59: 4310–4317.

2. Miller VL, Mekalanos JJ (1988) A novel suicide vector and its use in construction of insertion mutations: Osmoregulation of outer membrane proteins and virulence determinants in *Vibrio cholerae* requires toxR. J Bacteriol 170: 2575–2583.
